# Multiple Myeloma in Children and Young Adults: A Systematic Literature Review

**DOI:** 10.1007/s10238-026-02148-w

**Published:** 2026-05-21

**Authors:** Georgia Poultsaki, Theodoros P. Vassilakopoulos, Evangelos Terpos, Vassilios Papadakis

**Affiliations:** 1 Department of Pediatric Hematology - Oncology (TAO), ‘’Agia Sofia’’ Children’s Hospital Athens, Marianna V. Vardinoyanni Oncology Children’s Hospital, 8 Levadias Str, Goudi, Athens, 11527 Greece; 2 Department of Haematology and Bone Marrow Transplantation, National and Kapodistrian University of Athens (NKUA), Laikon General Hospital, 17 Agiou Thoma Str, Athens, 11527 Greece; 3 Department of Clinical Therapeutics, University of Athens School of Medicine, Alexandra General Hospital, 80 Vas. Sofias Ave, Athens, 11528 Greece; 4 School of Medicine, National and Kapodistrian University of Athens, Athens, Greece

**Keywords:** Pediatric Multiple Myeloma, Young Adults, Hematopoietic Stem Cell Transplantation, Extramedullary Disease, Plasmacytoma

## Abstract

Multiple myeloma (MM) is extremely rare in pediatric and young adult populations, and its clinical characteristics, therapeutic strategies, and outcomes remain poorly defined. This systematic review consolidates all published cases of MM in patients aged ≤ 25 years, aiming to define epidemiology, presentation, treatment, and theraupeutic outcomes. A systematic literature search was conducted in PubMed up to December 31, 2024, to identify case reports and series of MM in patients ≤ 25 years. Articles lacking patient-specific data, definitive diagnostic criteria, or full English text were excluded. Data on demographics, clinical features, cytogenetics, treatment regimens, and outcomes were extracted. Forty-two patients were included from 33 publications, with a median age of 17 years (range 8–25). Male predominance was noted (M: F = 1.47:1). Common presenting features included bone pain (42.9%) and neurological symptoms (21.4%). IgG myeloma was most frequent, followed by IgA, light-chain only and IgD subtypes. Plasmacytomas were observed in 61.9%, including 30.8% with extramedullary and 57.7% with paramedullary involvement. Cytogenetic abnormalities were detected in 75% of evaluable patients. First-line therapy varied by era, with regimens including conventional chemotherapy, novel agents, and hematopoietic stem cell transplantation (HSCT). Among the 16 patients with evaluable treatment response, the overall response rate to first-line therapy was 75%. Hematopoietic stem cell transplantation (HSCT) was performed in 10 patients and was associated with deeper treatment responses. Contemporary therapeutic regimens were generally well tolerated, and no therapy-related mortality was reported. MM in patients aged ≤ 25 years is a rare clinical entity that may present with distinctive features, including a high frequency of plasmacytomas and extramedullary disease. Modern treatment strategies incorporating novel agents and HSCT appear feasible and effective in this population. Further collaborative studies are required to better define the biological characteristics and optimal management of MM in very young patients.

**Supplementary Information:**

The online version contains supplementary material available at 10.1007/s10238-026-02148-w.

## Introduction

Multiple myeloma (MM) is a malignant neoplasm of plasma cells, accounting for approximately 1–1.8% of all cancers and representing the second most common hematologic malignancy. The estimated incidence in Europe ranges from 4.5 to 6.0 new cases per 100,000 individuals annually. Despite significant advancements in treatment over the past two decades, only 10–15% of MM patients achieve or surpass the life expectancy of the general population [1]. The median age at diagnosis is 70 years, with a minority of cases occurring in younger populations; specifically, 2% of cases are diagnosed in individuals under 40 years, and only 0.3% occur in those younger than 30 years [2].

Given the rarity of multiple myeloma (MM) in younger individuals, its clinical presentation, therapeutic strategies, and prognostic implications remain inadequately characterized. The majority of available data stem from case reports and small patient series. One of the most comprehensive sources of information is a multicenter retrospective study of 52 MM patients under 30 years of age diagnosed between 1986 and 2016 [2].

Additional studies have examined MM in younger populations. For example, a systematic review by Tanguay et al. [3] analyzed clinical manifestations and outcomes in young myeloma patients, while a large population-based analysis by Hira Mian et al. [4] reported disease characteristics and outcomes in 504 young patients treated in Canada. However, these studies primarily included broader young adult populations, whereas reports focusing specifically on very early-onset disease (≤ 25 years) remain extremely limited.

The objective of this systematic review is to address the existing gap in the literature since 2016 by collecting all newly reported cases and critically analyzing all available data. Furthermore, given the revision of diagnostic criteria in 2014 by the International Myeloma Working Group (IMWG) [5] and a more recent which is currently under publication, this review aims to assess potential changes in disease incidence and presentation. Additionally, this study aims to characterize the epidemiology, clinical and immunohematological features, therapeutic interventions, and patient outcomes based on a comprehensive review of all published case reports on MM in young patients. By systematically synthesizing available evidence, this work attempts to enhance the understanding of MM in very young populations and contribute to the refinement of diagnostic and therapeutic approaches in this unique patient subgroup.

## Methods

The present study systematically extracted and analyzed all confirmed cases of multiple myeloma (MM) reported in the literature. The age group of interest was defined as individuals ≤ 25 years at diagnosis. The literature search was conducted from March 1, 2024 through December 31, 2024, using PubMed to identify case reports and case series of patients within this age group.

The search strategy combined Medical Subject Headings (MeSH) and free-text keywords using Boolean operators (AND, OR). The main search string was: (“Myeloma, Plasma Cell“[Mesh] OR “multiple myeloma“[Title/Abstract] OR myeloma [Title/Abstract] OR plasmacytoma [Title/Abstract]) AND (“pediatric“[Title/Abstract] OR “young adult“[Title/Abstract] OR adolescent [Title/Abstract]) AND (“case report“[Publication Type] OR “case series“[Title/Abstract]). Filters were applied to include studies conducted in humans and published in English. The reference lists of the included articles were also manually screened to identify additional relevant studies. No date restrictions were applied.

Articles were excluded if they were not related to MM, represented duplicates, lacked definitive era-specific diagnostic criteria for MM, did not include patient-specific data, focused on monoclonal gammopathy of undetermined significance, asymptomatic multiple myeloma, or solitary plasmacytoma, were published in languages other than English and if not written in English, no comprehensive abstract was available.

Diagnosis of multiple myeloma was established according to the updated International Myeloma Working Group (IMWG) criteria. Cases were included if they met at least one of the following: clonal bone marrow plasma cells ≥ 10% or a biopsy-proven bony or extramedullary plasmacytoma, together with one or more myeloma-defining events. These included: evidence of end-organ damage attributable to the plasma cell disorder (hypercalcemia, renal insufficiency, anemia, or bone lesions) or biomarkers of malignancy (clonal bone marrow plasma cells ≥ 60%, involved/uninvolved serum free light chain ratio ≥ 100, or > 1 focal lesion on MRI). Each case was individually assessed to confirm that it met the diagnostic standards at the time of publication. Disease staging was performed according to the International Staging System (ISS) [6]. The specific IMWG diagnostic criteria fulfilled for each patient are summarized in Supplementary Table S1.

Two independent researchers developed and performed the literature search. Titles and abstracts of all retrieved articles were screened for eligibility, and full texts were reviewed when inclusion or exclusion could not be determined from the abstract alone. Any disagreements were resolved by consensus or, if necessary, by a third researcher. The final cohort was compiled from individual case reports available in the published literature [7–40].

## Results

### Disease characteristics at diagnosis

**Fig. 1:**
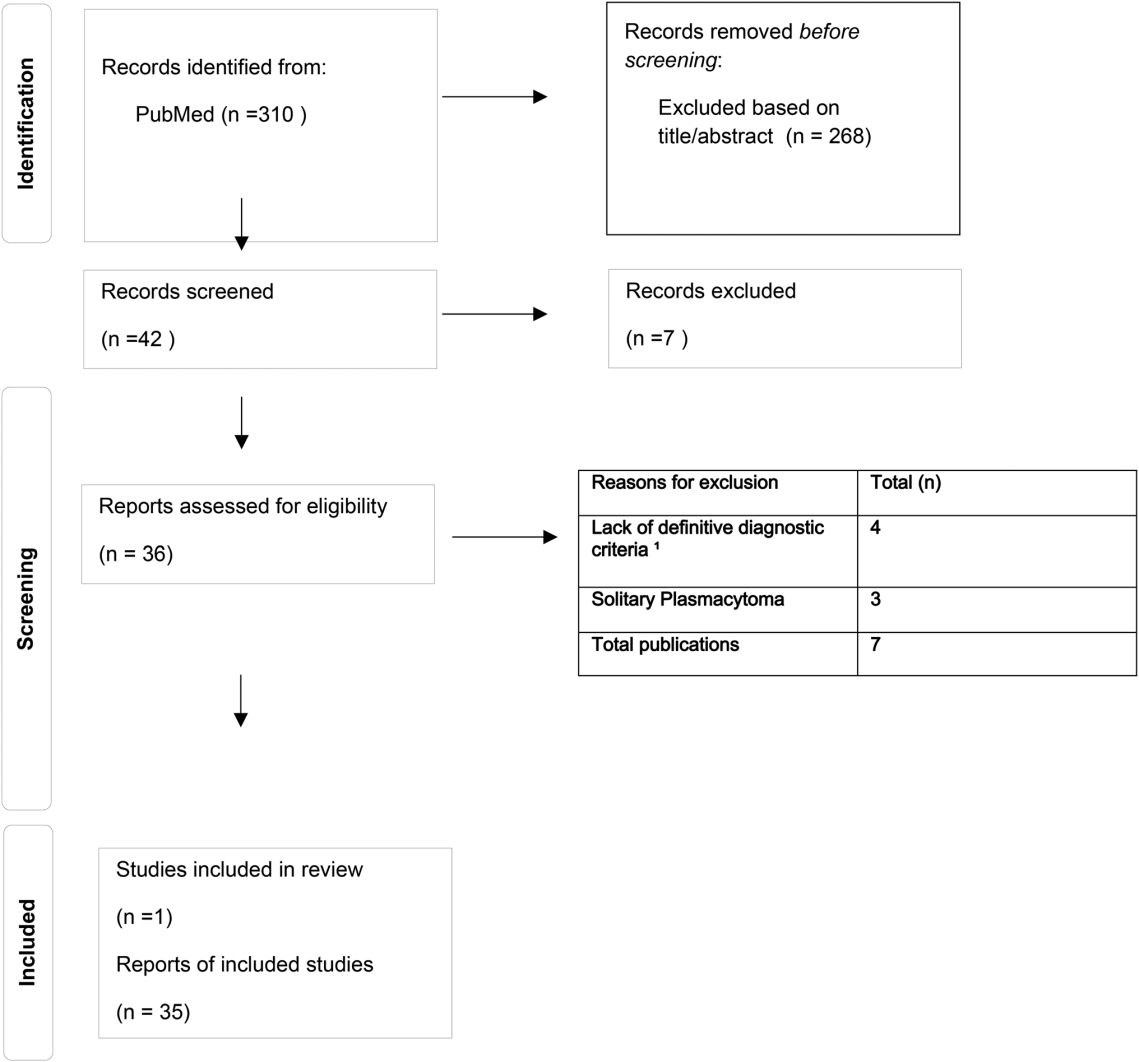
Adapted Preferred Reporting Items for Systematic Reviews and Meta-Analyses (PRISMA) flow diagram, showing the number of papers identified in the initial search, numbers excluded for various reasons and the final numbers of papers in each category studied which is the basis of the data presented.¹ Studies that did not apply standard multiple myeloma diagnostic criteria

Table. 1 A total of 51 patients were identified through literature search via PubMed. Of these, 9 were excluded from further analysis due to a diagnosis other than MM. The PRISMA flow diagram is shown in Fig. 1. Detailed reference information for each included patient is provided in Supplementary Table 2. The diagnosis period ranged from 1956 to 2024, with most patients diagnosed between 2011 and 2024 (20/42, 47.6%). Of the 42 included patients, 22 were diagnosed before 2014 and 20 after the revision of the IMWG diagnostic criteria. Patient characteristics are summarized in Table 2. The median age at diagnosis for the remaining 42 patients was 17 (range 8–25) years. Among these, 28 patients were 20 years or younger and 6 were under 10 years of age at the time of diagnosis. A male predominance was observed, with a male-to-female ratio of 1.47:1 (25 males to 17 females). The sex distribution at diagnosis showed no significant variation across age groups.

**Table 1:** List of Abbreviations

Abbreviation	Full Term
AL	Amyloid Light-chain
Allo-SCT	Allogeneic Stem Cell Transplantation
ASCT	Autologous Stem Cell Transplantation
BCNU	Carmustine (Bis-chloroethylnitrosourea)
CHOP	Cyclophosphamide, Doxorubicin, Vincristine, Prednisone
CI	Confidence Interval
COP	Cyclophosphamide, Vincristine, Prednisone
CR	Complete Response
CyBorD	Cyclophosphamide, Bortezomib, Dexamethasone
Dara-VRD	Daratumumab, Bortezomib, Lenalidomide, Dexamethasone
HIV	Human Immunodeficiency Virus
HSCT	Hematopoietic Stem Cell Transplantation
IgA / IgD / IgG	Immunoglobulin A / D / G
IMiDs	Immunomodulatory Drugs
IMWG	International Myeloma Working Group
ISS	International Staging System
MM	Multiple Myeloma
MP	Melphalan, Prednisone
MPC-B	Melphalan, Prednisone, Cyclophosphamide, BCNU
OS	Overall Survival
PD	Progressive Disease
PFS	Progression-Free Survival
PI(s)	Proteasome Inhibitor(s)
PR	Partial Response
SPSS	Statistical Package for the Social Sciences
TD	Thalidomide, Dexamethasone
VAD	Vincristine, Adriamycin (Doxorubicin), Dexamethasone
VBCMP	Vincristine, BCNU, Cyclophosphamide, Melphalan, Prednisone
VCDP	Vincristine, Cyclophosphamide, Doxorubicin, Prednisone
VD	Bortezomib, Dexamethasone
VDCEP	Bortezomib, Dexamethasone, Cyclophosphamide, Etoposide, Cisplatin
VGPR	Very Good Partial Response
VRD	Bortezomib, Lenalidomide, Dexamethasone

**Table 2:** Clinical and biological characteristics at diagnosis

Characteristic	Frequency *N*%
Diagnosis period 1956–1999 2000–2005 2006–2010 2011–2024	16/42(38.1%) 3/42(7.1%) 3/42(7.1%) 20/42(47.6%)
Sex Male Female	25/42(59.5%) 17/42(40.5%)
**Median age (range)** **20-25y** **10-20y** **< 10y**	17(8.0–25.0) 14/42(32.6%) 22/42(52.4%) 6/42(14.3%)
**Symptoms** Weight loss Neurological Bleeding Fatigue Fever Bone pain	5/42(11.9%) 9/42(21.4%) 4/42(9.5%) 1/42(2.4%) 5/42(11.9%) 19/42(42.9%)
**Plasmacytomas** Extramedullary Paramedullary Extramedullary& Paramedullary	26/42(61.9%) 8/26(30.8%) 15/26(57.7%) 3/26(11.5%)
**CRAB criteria** Hemoglobin < 10 g/dL Creatinine > 2 mg/dL Calcium > 11 mg/dL Lytic bone lesions	17/38(44.7%) 5/30(16.7%) 3/32(9.4%) 32/38(84.2%)
**Proteinuria**	31(86.1%)
**Albumin < 3.5 g/dL**	8/20(40.0%)
**Β2 microglobulin ≥ 3.5 mg/dL**	2/11(18.2%)
**Μ protein** IgG IgA IgD Light chain only Non secretory	18/38(47.4%) 12/38(31.6%) 1/38(2.6%) 6/38(15.8%) 1/38(2.6%)
**Light chain type**κλ Light chain uknown	17/37(45.9%) 15/37(40.5%) 5/37(13.5%)
**ISS stage** I II III	8/14(57.1%) 2/14(14.3%) 4/14(28.6%)

a. Percentages are calculated based on available data for each variable.b. ISS = International Staging System for multiple myeloma.c. Some categories exceed 100% because patients may present with more than one feature (e.g., symptoms, plasmacytomas).

The most prevalent symptoms at diagnosis were bone pain (42.9%) and neurological symptoms (21.4%). IgG was the predominant M-protein subtype, followed by IgA, whereas IgD was observed in only one case. Light chain myeloma was identified in 15.8% of patients, while one patient was diagnosed with non-secretory myeloma. At diagnosis, 26 out of 42 patients (61.9%) presented with plasmacytomas. The most common sites of plasmacytoma involvement were the bones (57.7%), with the spine and the skull regions being primarily affected. Both extramedullary and paramedullary disease were observed in 3 of 26 patients (11.5%), and a rare site of involvement included the breasts, which was identified in a 13-year-old patient [7]. Lambda light chain amyloidosis was diagnosed in a 21-year-old patient through myocardial biopsy [8]. CRAB criteria were frequently present, with anemia (45%) and lytic bone lesions (84%) being most common. ISS stage was reported in 14 patients, with stage I being most frequent (57%).

Cytogenetic data was available for 12 patients (28.6%). Chromosomal abnormalities were detected in 9 of these patients (75%), as detailed in Table 3.

**Table 3:** High-risk FISH features

Chromosomal alteration	Genetic involvement	Frequency
t(11;14)	IGH/CCND1 rearrangement	1/12
t(8;14)	MYC activation	1/12
(mut)1q21	CKS1B mutation	1/12
MYC rearrangement with unidentified partner	MYC activation	2/12
del(22q12)	EWSR1 deletion	1/12
i(1q)	CKS1B amplification	1/12
(del)1p11.2 & del(13q14)	RB1deletion	1/12
point mutation in exon 4 c.215 C > G): p.Pro72Arg	TP53 mutation	1/12

FISH: fluorescence in situ hybridization. del = deletion; mut = mutation; i = isochromosome. IGH = immunoglobulin heavy chain gene; CCND1 = cyclin D1; MYC = transcription factor and oncogene; CKS1B = cell cycle regulatory protein; EWSR1 = Ewing sarcoma RNA-binding protein 1; RB1 = retinoblastoma tumor suppressor gene; TP53 = p53 tumor suppressor gene.

## Treatment

The choice of first-line treatment depended on the time period of diagnosis. Patients before 2006 received conventional chemotherapy, i.e. COP (Cyclophosphamide, Vincristine, Prednisone), CHOP (Cyclophosphamide, Doxorubicin, Vincristine, Prednisone), VAD/MP (Vincristine, Adriamycin, Dexamethasone/Melphalan, Prednisone), VCDP (Vincristine, Cyclophosphamide, Doxorubicin, Prednisone), VBCMP (Vincristine, BCNU, Cyclophosphamide, Melphalan, Prednisone) and MPC-B (Melphalan, Prednisone, Cyclophosphamide, BCNU) regimens. Patients treated before 2006 were excluded from the analysis due to limited and low-quality data reported in these cases. A total of 24 patients were diagnosed after 2006, following the introduction of novel therapeutic agents for multiple myeloma. Among these, two patients (aged 8 and 25 years) succumbed prior to initiating treatment: the first due to septic shock associated with HIV-induced immunodeficiency [7], and the second due to acute kidney injury complicated by concomitant acute pulmonary edema [8]. Treatment response data were unavailable for six patients who received regimens including bortezomib or immunomodulatory drug (IMiD)-based regimens, i.e. VDCEP (Bortezomib, Dexamethasone, Cyclophosphamide, Etoposide, Cisplatin), TD (Thalidomide, Dexamethasone), CyBorD (Cyclophosphamide, Bortezomib, Dexamethasone), VRD (Bortezomib, Lenalidomide, Dexamethasone), and Dara-VRD (Daratumumab, Bortezomib, Lenalidomide, Dexamethasone). Consequently, treatment response to first-line therapy was evaluable in 16 patients. Abbreviations for all regimens are listed in Table 1.

Of the 16 evaluable patients, five (31.3%) received a triplet regimen consisting of a proteasome inhibitor (PI), an immunomodulatory drug (IMiD), and corticosteroids. Three patients (18.8%) were treated with doublet regimens, four (25%) received combinations of novel agents with conventional chemotherapy, and the remaining four (25%) were treated with conventional chemotherapy alone. The most commonly used regimens were VRD (bortezomib/lenalidomide/dexamethasone) and VAD (vincristine/doxorubicin/dexamethasone). Bortezomib/dexamethasone (VD), with or without additional agents such as thalidomide, lenalidomide, doxorubicin, or cyclophosphamide, was administered in 11 patients. Radiotherapy was used in five patients, including one case for palliative purposes.

Upfront hematopoietic stem cell transplantation (HSCT) was performed in 10 of the 16 patients. Five underwent a single autologous stem cell transplantation (ASCT), while two patients received tandem ASCT, one of whom subsequently proceeded to allogeneic transplantation. Additionally, three patients received upfront allogeneic stem cell transplantation without prior ASCT. No specific indications for tandem autologous stem cell transplantation (ASCT) or allogeneic SCT were reported in the reviewed cases. First-line regimens administered and type of HSCT are summarized in Table 4;

## Treatment Response

Treatment response to first-line therapy was evaluable in all 16 patients. Among them, 7 (43.8%) achieved complete response (CR), 2 (12.5%) very good partial response (VGPR), 3 (18.7%) partial response (PR), 1 (6.3%) had stable disease (SD), and 3 (18.7%) experienced progressive disease (PD) requiring second-line therapy, resulting in an overall response rate (ORR) of 75%. First-line treatment regimens and response outcomes are described in Table 4; For the patient treated with CyBorD, the overall response rate (ORR) was 33% (1/3); the single non-responder represents an unexplained outlier, as no treatment- or disease-related factors could clearly account for the lack of response.

**Table 4:** Treatment regimens and response outcomes in evaluable patients (*n* = 16)

**Induction regimen**	Frequency*N*(%)	ORR*n* (%)
VRD VAD VD CyBord VTD CTD	4(25) 4(25) 3(18.7) 3(18.7) 1(6.3) 1(6.3)	2(50) 4(100) 3(100) 1(33.3) 1(100) 1(100)
**HSCT** ASCT Tandem ASCT Allo-HSCT	10(62.5) 5(50) 2(20) 5(50)	
**Maintenance therapy**	3(18.8)	
**Radiotherapy**	5(31.3)	
**Response to first-line therapy** CR VGPR PR Stable disease Progressive disease	7(43.8) 2(12.5) 3(18.7) 1(6.3) 3(18.7)	
**Response to HSCT** CR VGPR PR	7(70) 2(20) 1(10)	10(100)

Abbreviations are listed in Table 1**.**

Patients who died prior to therapy or lacked response data were excluded from ORR analysis.Patients who died prior to therapy or lacked response data were excluded from ORR analysis.

Ten patients proceeded to hematopoietic stem cell transplantation (HSCT). Prior to HSCT, 4/10 (40%) were in CR, 2/10 (20%) in VGPR, 3/10 (30%) in PR, and 1/10 (10%) had PD. After HSCT, deeper responses were observed: 7/10 (70%) achieved CR, 2/10 (20%) VGPR and 1/10 (10%) PR, resulting in an ORR of 100%. Among patients who underwent allogeneic HSCT, relapse occurred in three cases after transplantation.

Six patients did not receive HSCT: one patient from Jamaica due to local economic constraints and lack of a matched related donor [11], and another due to worsening cardiac amyloidosis. Three patients died—two from disease progression after multiple relapses, and one from AL amyloidosis with cardiac involvement [8]. Table 6 summarizes induction regimens, treatment responses, second-line therapies, HSCT conditioning, maintenance therapy, relapse events as well as follow-up duration for the 16 MM patients for whom treatment outcome data were available. This overview highlights the heterogeneity in therapeutic approaches and outcomes in this young patient population.

Treatment responses were further analyzed according to age groups (< 10, 10–20, 20–25 years) to explore potential differences in outcomes. Descriptive analysis of treatment outcomes by age group showed that most patients achieved CR or VGPR regardless of age. Two deaths occurred in the 10–20 year group and one in the 20–25 year group. Given the small sample size, no definitive conclusions on age-related differences can be drawn.

## Discussion

The current study has analyzed the clinicopathological characteristics of extremely young MM patients to identify subtle differences in clinical presentation compared to the well-documented elderly MM population. Key clinical characteristics from prior studies, summarized in Table 5, highlight areas of concordance and distinction. Notably, the findings reinforce previously reported trends, including a male predominance and a significant proportion of young patients diagnosed at ISS stage I. Additionally, consistent with prior literature, IgG emerged as the most frequently observed M protein subtype in this population, as in the elderly patients. In terms of the initial clinical presentation, pediatric patients most commonly exhibit anemia and bone lesions, as observed in the adult population.

Some notable differences were revealed in this review. A relatively large proportion (31.6%) had IgA myeloma, compared to 17% and 11% among patients younger than 40, and 18% among those aged 30 years or younger in other studies [2, 40, 41]. Interestingly, plasmacytomas were observed in 61.9% of patients in our cohort, with 30.8% extramedullary, 57.7% paramedullary, and 11.5% showing both types. These proportions are substantially higher than those reported in adult populations, where the overall incidence ranges from 7% to 20%, with 17.6% skeletal and 1.9% extramedullary [44]. This suggests that very young multiple myeloma patients may have a greater predisposition to plasmacytoma formation, particularly involving extramedullary sites, which could reflect distinct disease biology in this age group.

The introduction of novel therapeutic agents and hematopoietic stem cell transplantation (HSCT) has improved overall response rates, highlighting the feasibility of intensive treatment strategies in young patients. Importantly, no therapy-related mortality was reported, supporting the safety of contemporary regimens when carefully managed.

Despite these findings, the retrospective nature and limited sample size of the study constrain generalizability. Publication and selection bias may also influence the results, as studies reporting favorable outcomes are more likely to be published, potentially overestimating treatment effectiveness. Future studies incorporating larger, prospectively collected cohorts or unpublished datasets could help validate these observations and further clarify disease characteristics in this population.

A major strength of this study is the comprehensive assessment of a wide range of clinicopathological features for patients aged 25 or younger, representing a rare and understudied subgroup of MM. To our knowledge, this is the first systematic review focusing on such a young cohort, including individuals from diverse national backgrounds. It is important to acknowledge that the proportion of patients under 10 years old in our cohort (6/42, 14.3%) may not reflect the true population distribution, as case reports are inherently biased toward unusual or atypical presentations. Similarly, conclusions regarding treatment efficacy and outcomes of hematopoietic stem cell transplantation (HSCT) should be interpreted cautiously, given the small number of evaluable patients (n=16) and relatively short follow-up. These observations are therefore exploratory and hypothesis-generating, highlighting the need for larger, prospectively collected datasets to validate findings in this rare and very young MM population.

In conclusion, MM in very young patients presents unique clinical and therapeutic challenges. The integration of novel agents and HSCT appears safe and effective, and the high frequency of extramedullary involvement and IgA subtype suggests potential differences in disease biology compared to older patients. Future research should aim to identify molecular biomarkers predictive of treatment response and to develop personalized therapeutic approaches tailored to this distinct patient population.

**Table 5:** Comparison of patient characteristics across several MM cohort studies in children and adults

Characteristic	Present study *N* = 42	Shin et al. 2017 [41] *N* = 32	Yanamandraet al. 2018 [40] *N* = 40	Jurczyszyn et al. 2019 [2] *N* = 52	Lu et al. 2016[43] *N* = 746
**Country**	Europe, Africa, India, Brazil, Mexico, USA, Indonesia, Vietnam, Japan	South Korea	India	Europe, USA, Brazil, Hong Kong	China
**Years of diagnosis**	1956–2024	2000–2015	2010–2015	1989–2016	2008–2011
**Patients’ age (years)**	≤ 25	≤ 40	≤ 40	≤ 30	≥ 50
**Median age (range)**	17(8–25)	37(17–40)	38(15–39)	28(8–30)	62(50–88)
**Included patients < 30y**	42	4	5	52	0
**Male/Female**	25/17	19/13	26/14	35/17	457/289
**ISS stage**					
Stage I	57.1%	32%	13%	68%	17%
Stage II	14.3%	48%	17%	15%	32%
Stage III	28.6%	20%	70%	17%	51%
**M protein subtype**					
IgG	47.4%	47%	76%	55%	46%
IgA	31.6%	17%	11%	18%	19%
Light chain	15.8%	30%	11%	22%	33%
**Hemoglobin < 10 mg/dL**	44.7%	29%	53%	30%	61%
**Calcium > 11 mg/dL**	9.4%	28%	24%	14%	NA
**Bone disease**	84.2%	87%	59%	82%	83%
**Creatinine > 2 mg/dL**	16.7%	13%	30%	18%	25%%
**Upfront HSCT**	62.5%	62%	NA	62%	NA

Abbreviations are listed in Table 1.

Patients who died prior to therapy or lacked response data were excluded from ORR analysis

Categories may overlap (e.g., patients receiving tandem ASCT or subsequent allogeneic transplantation).

## Supplementary Information

Below is the link to the electronic supplementary material.

Supplementary material 1 (DOCX 40.4 kb)

## Data Availability

The data supporting the findings of this study are available from previously published case reports, which are referenced in this manuscript. No new data were generated in the course of this study. All sources are cited in the reference list.
