# Supplementary material for: Multiple Myeloma in Children and Young Adults: A Systematic Literature Review
Source: Clin Exp Med. 2026 May 21;26(1):271. doi: 10.1007/s10238-026-02148-w (PMC13375675; doi:10.1007/s10238-026-02148-w)
Supplement: Supplementary file 1 — Supplementary material 1 (DOCX 40.4 kb) [file 10238_2026_2148_MOESM1_ESM.docx]

**Supplementary Table S1. Fulfillment of International Myeloma Working Group (IMWG) diagnostic criteria for each included case.**

| **Case No** | **Ref No** | **Clonal bone marrow plasma cells** | **Plasmacytomas/ Extramedullary disease** | **CRAB features** | **Biomarkers** | **Notes** |
| --- | --- | --- | --- | --- | --- | --- |
| 1 | 7 | 90% | Breast and abdomen | Hb 11.6 g/dL | NP | NP |
| 2 | 8 | 80% | NP | NP | Free light chain ratio (FLC)**≥**100 | Amyloidosis with heart involvement |
| 3 | 9 | Bone marrow biopsy: sheets of large atypical plasma cells CD138+,CD56+ | NP | Hb 5g/dL, Ca 15 mg/dL, Lytic lesions: skull, scapula, humerus, vertebrae, ribs, pelvis bones | κ/λ ratio:0.007 | NP |
| 4 | 10 | 12% | NP | Hb 9.6 g/dL | NP | NP |
| 5 | 11 | 2% | Cervical lymph node mass | Lytic lesions: skull, femur, vertebra compression fracture | NP | NP |
| 6 | 12 | 43% | FNA: Retroperitoneal lymph nodes involvement | Hb 5.8 g/dL, Skull lytic lesions | NP | NP |
| 7 | 13 | NP | Femoral and clavicle | Hb 7g/dL, Lytic lesions: skull, ribs, spine, facial and long bones, clavicles, shoulders | NP | NP |
| 8 | 14 | 95% | Ethmoidal | Hb 7.9 g/dL, Lytic lesions: skull, humerus, radius, clavicle, ribcage, pelvis, spine, tibia, fibula, femur | NP | NP |
| 9 | 15 | NP | Skull | Lytic lesions: skull, ribs | NP | NP |
| 10 | 15 | NP | Tibia | Lytic lesions: tibia with a pathological fracture | NP | NP |
| 11 | 16 | 90% | NP | Hb 8.1 g/dL, Creatinine 2.8 mg/dL | NP | Light chain cast nephropathy |
| 12 | 17 | 30% | T7 vertebra | Lytic lesions: skull, ischia | NP | NP |
| 13 | 17 | Plasmacytosis | T12 vertebra | Lytic lesions: skull and destruction of the mandible | NP | NP |
| 14 | 18 | NP | Orbital, Clavicle | Hb 10g/dL, Lytic lesion: Frontal bone, clavicle | NP | NP |
| 15 | 19 | NP | Ocipital, Tibia | Lytic lesions: skull, femur, tibia, hamate bone | NP | NP |
| 16 | 19 | NP | Pharyngeal | Lytic lesions: ribs, calvarium | NP | NP |
| 17 | 19 | NP | Occipital | Skull lytic lesions | NP | NP |
| 18 | 20 | NP | L3 vertebra | Lytic lesions: rib, vertebra | NP | NP |
| 19 | 21 | NP | Chest wall | Lytic lesions: ribs, scapula, femur | NP | NP |
| 20 | 21 | 68% | NP | Hb 5.3 g/dL, Creatinine 2.2 mg/dL, Ca 13.9 mg/dL | NP | Circulating blasts |
| 21 | 22 | 6.5% | NP | Hb 10.1 g/dL, Multiple thoracic compression fractures | FLC ratio 99,40 g/L | NP |
| 22 | 23 | 3% | Ilium | Hb 10.9 g/dL, Lytic lesions: pubis, ilium | NP | NP |
| 23 | 24 | 30% | NP | Hb 7.3 g/dL | Free κ/λ ratio 0,15 | NP |
| 24 | 25 | NP | Soft tissue sternal mass | Several soft tissue masses with destruction on skull base and mandible | FLC ratio**≥**100 | NP |
| 25 | 26 | 60% | NP | Hb 5.3 g/dL, Creatinine 2.2 mg/dL, Lytic lesions: vertebral bodies and skull bone | NP | NP |
| 26 | 27 | Flow cytometry: CD138+, bright CD 38, CD 19-, dim CD45, monotypic λ light chain restriction | NP | Hb 9.4 g/dL, Creatinine 1.63 mg/dL, Ca 16.6 mg/dL | NP | NP |
| 27 | 27 | NP | L3 Vertebra | Lytic lesions: vertebrae, ribs, calvarium | NP | NP |
| 28 | 28 | 5% | Tissue biopsies dystrophic PC, MI 15+ with no monoclonality | Hb 10.6 g/dL, Lytic lesions: femur, sternum, vertebra | FLC ratio**≥**100 | History of JMML at the age of 4 months |
| 29 | 29 | Normal | Skull base mass | Hb 10 g/dL, Lytic lesions: ribs, vertebra | NP | NP |
| 30 | 30 | NP | NP | Anemia, | NP | NP |
| 31 | 31 | 10% | NP | Anemia, Hip lytic lesion | NP | NP |
| 32 | 32 | 43% | NP | Hb 9.2 g/dL | NP | NP |
| 33 | 33 | 30% | Left femur | Femur fracture | NP | NP |
| 34 | 34 | 3% | Soft tissue mass, Right shoulder | Lytic lesions | NP | NP |
| 35 | 34 | 90% | NP | Creatinine 2mg/dL, Lytic lesions | NP | NP |
| 36 | 34 | 2% | Multiple soft tissue masses | Lytic lesions | NP | NP |
| 37 | 35 | Normal | C7 vertebra | Lytic lesions: spine, rib | NP | NP |
| 38 | 36 | 30% | Occipital mass, cervical lymph node | Lytic lesions: long bones, sacrum, vertebrae | NP | NP |
| 39 | 37 | 10% | Right humerus, Submandibular lymph node | Lytic lesion and fraction of the right humerus | NP | NP |
| 40 | 38 | 55.2% | NP | Hb 7,5 g/dL, Lytic lesions: vertebrae, sternum, scapula, humerus, ribs | NP | NP |
| 41 | 39 | Flow cytometry: CD138+, kappa light chain restriction plasma cells | NP | Anemia, Creatinine 2.3 mg/dL | NP | NP |
| 42 | 40 | 30% | T6 vertebra mass | Anemia, Lytic lesions: humerus, ribs, ischium and pathologic compression fracture of T6 vertebrae | NP | NP |

a. NP (not present) indicates the absence of the corresponding feature in the patient.
b. CRAB: hyperCalcemia (Ca >11 mg/dL), Renal insufficiency (Creatinine >2 mg/dL), Anemia (Hb <10 g/dL), Bone lesions (lytic lesions or fractures).

c. Plasmacytomas/Extramedullary disease: sites of plasmacytoma or extramedullary involvement.

d. Biomarkers: relevant laboratory or molecular findings.

e. Notes: additional clinical information.

f. JMML= juvenile myelomonocytic leukemia.

**Supplementary Table 2. Case-by-case reference information for the 42 multiple myeloma patients ≤25 years.**

| **Case No** | **Ref No** | **First Author** | **Year** | **Country** | **PMID** | **DOI** | **Age** | **Sex** |
| --- | --- | --- | --- | --- | --- | --- | --- | --- |
| 1 | 7 | Maeda | 1967 | USA | **4126526** | [10.1093/ajcp/60.4.552](https://doi.org/10.1093/ajcp/60.4.552) | 13 | F |
| 2 | 8 | Camus | 2019 | France | **31183088** | [10.1002/ccr3.2165](https://doi.org/10.1002/ccr3.2165) | 21 | M |
| 3 | 9 | Radhakrishnan | 2017 | India | **27820125** | [10.1097/MPH.0000000000000684](https://doi.org/10.1097/mph.0000000000000684) | 8 | M |
| 4 | 10 | Cong | 2024 | Indonesia | NR | [10.15562/bmj.v13i3.5281](https://doi.org/10.15562/bmj.v13i3.5281) | 12 | F |
| 5 | 11 | Brady-West | 2009 | Jamaica | **19715046** | [10.1016/s00279684(15)31012-9](https://doi.org/10.1016/s0027-9684(15)31012-9) | 16 | M |
| 6 | 12 | Mohanty | 2004 | India | **15349993** | [10.1002/dc.20039](https://doi.org/10.1002/dc.20039) | 8 | M |
| 7 | 13 | Kaste | 1992 | Brazil | **1523057** | [10.1007/BF02019864](https://doi.org/10.1007/bf02019864) | 15 | M |
| 8 | 14 | García-Álvarez | 2012 | Mexico | **22670629** | [10.2350/12-05-1187-CR.1](https://doi.org/10.2350/12-05-1187-cr.1) | 9 | F |
| 9 | 15 | Ishida | 1995 | USA | **7709252** | [10.1007/BF02425947](https://doi.org/10.1007/bf02425947) | 23 | F |
| 10 | 15 | Ishida | 1995 | USA | **7709252** | [10.1007/BF02425947](https://doi.org/10.1007/bf02425947) | 21 | M |
| 11 | 16 | Mufti | 2018 | USA | **32300415** | [10.14740/jh380w](https://doi.org/10.14740/jh380w) | 20 | F |
| 12 | 17 | Geetha | 1999 | India | **NR** | [10.1259/bjr.72.862.10673955](https://doi.org/10.1259/bjr.72.862.10673955) | 20 | F |
| 13 | 17 | Geetha | 1999 | India | **NR** | [10.1259/bjr.72.862.10673955](https://doi.org/10.1259/bjr.72.862.10673955) | 18 | F |
| 14 | 18 | Levin | 1977 | USA | **849187** | [10.1001/archopht.1977.04450040108016](https://doi.org/10.1001/archopht.1977.04450040108016) | 18 | F |
| 15 | 19 | Hewell | 1976 | USA | **1259287** | [10.7326/0003-4819-84-4-441](https://doi.org/10.7326/0003-4819-84-4-441) | 17 | M |
| 16 | 19 | Hewell | 1976 | USA | **1259287** | [10.7326/0003-4819-84-4-441](https://doi.org/10.7326/0003-4819-84-4-441) | 22 | M |
| 17 | 19 | Hewell | 1976 | USA | **1259287** | [10.7326/0003-4819-84-4-441](https://doi.org/10.7326/0003-4819-84-4-441) | 20 | M |
| 18 | 20 | Ronald | 2024 | Kenya | **38645602** | [10.1002/ccr3.8801](https://doi.org/10.1002/ccr3.8801) | 9 | M |
| 19 | 21 | Davidow | 2019 | USA | **30393949** | [10.1002/pbc.27528](https://doi.org/10.1002/pbc.27528) | 12 | F |
| 20 | 21 | Davidow | 2019 | USA | **30393949** | [10.1002/pbc.27528](https://doi.org/10.1002/pbc.27528) | 16 | M |
| 21 | 22 | Wang | 2021 | Spain | **33869065** | 1[0.3389/fonc.2021.662169](https://doi.org/10.3389/fonc.2021.662169) | 14 | M |
| 22 | 23 | Man | 2019 | Vietnam | **31685790** | [10.12659/AJCR.917670](https://doi.org/10.12659/ajcr.917670) | 17 | F |
| 23 | 24 | Merich | 2023 | Algeria | **NR** | 10.35248/23298790.23.11.547 | 24 | F |
| 24 | 25 | Lee | 2020 | USA | **32099696** | [10.1155/2020/1375174](https://doi.org/10.1155/2020/1375174) | 24 | F |
| 25 | 26 | Crusoe | 2015 | Brazil | **25441111** | [10.1016/j.clml.2014.08.004](https://doi.org/10.1016/j.clml.2014.08.004) | 8 | M |
| 26 | 27 | Yohe | 2017 | USA | **28719511** | [10.1097/MPH.0000000000000907](https://doi.org/10.1097/mph.0000000000000907) | 21 | M |
| 27 | 27 | Yohe | 2017 | USA | **28719511** | [10.1097/MPH.0000000000000907](https://doi.org/10.1097/mph.0000000000000907) | 14 | M |
| 28 | 28 | Auger | 2009 | France | **NR** | [10.1182/blood.V114.22.4957.4957](https://doi.org/10.1182/blood.V114.22.4957.4957) | 10 | M |
| 29 | 29 | Lesar | 2024 | South Africa | [39114744](https://pubmed.ncbi.nlm.nih.gov/39114744/) | [10.4102/sajr.v28i1.2883](https://doi.org/10.4102/sajr.v28i1.2883) | 21 | F |
| 30 | 30 | Hathsuse | 2005 | Japan | **NR** | [10.11406/rinketsu.56.428](https://doi.org/10.11406/rinketsu.56.428) | 14 | M |
| 31 | 31 | Mohsen | 2023 | Egypt | **NR** | [10.1016/S21522650(23)01398-8](https://doi.org/10.1016/S2152-2650(23)01398-8) | 16 | M |
| 32 | 32 | Munoz | 2012 | USA | **NR** | 10.1182/blood-2011-06-362905 | 23 | F |
| 33 | 33 | Badwey | 1993 | USA | **8358931** | NR | 25 | F |
| 34 | 34 | Blade | 1996 | USA | **8678716** | [10.1001/archinte.1996.00440120125014](https://doi.org/10.1001/archinte.1996.00440120125014) | 19 | F |
| 35 | 34 | Blade | 1996 | USA | **8678716** | [10.1001/archinte.1996.00440120125014](https://doi.org/10.1001/archinte.1996.00440120125014) | 23 | M |
| 36 | 34 | Blade | 1996 | USA | **8678716** | [10.1001/archinte.1996.00440120125014](https://doi.org/10.1001/archinte.1996.00440120125014) | 20 | M |
| 37 | 35 | Rapoport | 1990 | USA | **NR** | [10.1016/00029343(90)90229-7](https://doi.org/10.1016/0002-9343(90)90229-7) | 15 | M |
| 38 | 36 | Powari | 2001 | India | **11391729** | [10.1002/ajh.1117](https://doi.org/10.1002/ajh.1117) | 13 | F |
| 39 | 37 | Powari | 2002 | India | **NR** | 10.104[10.1046/j.13652303.2002.t01-3-00381.x](https://doi.org/10.1046/j.1365-2303.2002.t01-3-00381.x) | 13 | M |
| 40 | 38 | Li | 2009 | China | **NR** | [https:10.1016/j.leukres.2008.08.013](https://doi.org/10.1016/j.leukres.2008.08.013) | 23 | M |
| 41 | 39 | Akhtar | 2017 | Pakistan | **NR** | NR | 25 | M |
| 42 | 40 | Combe | 2023 | USA | **NR** | NR | 23 | M |

Abbreviations: PMID, PubMed Identifier; DOI, Digital Object Identifier; M, male; F, female; NR, not repored.
